# Gaze and Event Tracking for Evaluation of Recommendation-Driven Purchase

**DOI:** 10.3390/s21041381

**Published:** 2021-02-16

**Authors:** Piotr Sulikowski, Tomasz Zdziebko, Kristof Coussement, Krzysztof Dyczkowski, Krzysztof Kluza, Karina Sachpazidu-Wójcicka

**Affiliations:** 1 Faculty of Information Technology and Computer Science, West Pomeranian University of Technology, ul. Żołnierska 49, 71-210 Szczecin, Poland; 2 Faculty of Economics, Finance and Management, University of Szczecin, ul. Adama Mickiewicza 64, 71-101 Szczecin, Poland; tomasz.zdziebko@usz.edu.pl; 3 IÉSEG School of Management, 3 Rue de la Digue, F-59000 Lille, France; k.coussement@ieseg.fr; 4 LEM-CNRS 9221, 3 Rue de la Digue, F-59000 Lille, France; 5 Faculty of Mathematics and Computer Science, Adam Mickiewicz University, ul. Uniwersytetu Poznańskiego 4, 61-614 Poznań, Poland; chris@amu.edu.pl; 6 Faculty of Electrical Engineering, Automatics, Computer Science and Biomedical Engineering, AGH University of Science and Technology, al. Adama Mickiewicza 30, 30-059 Kraków, Poland; kluza@agh.edu.pl; 7 Faculty of Business and Management, Wrocław University of Economics and Business, ul. Komandorska 118/120, 53-345 Wrocław, Poland; karina.sachpazidu-wojcicka@ue.wroc.pl

**Keywords:** e-commerce, human activity recognition, human–computer interaction, eye tracking, event tracking, mouse tracking, recommender systems, visual, layout, implicit feedback

## Abstract

Recommendation systems play an important role in e-commerce turnover by presenting personalized recommendations. Due to the vast amount of marketing content online, users are less susceptible to these suggestions. In addition to the accuracy of a recommendation, its presentation, layout, and other visual aspects can improve its effectiveness. This study evaluates the visual aspects of recommender interfaces. Vertical and horizontal recommendation layouts are tested, along with different visual intensity levels of item presentation, and conclusions obtained with a number of popular machine learning methods are discussed. Results from the implicit feedback study of the effectiveness of recommending interfaces for four major e-commerce websites are presented. Two different methods of observing user behavior were used, i.e., eye-tracking and document object model (DOM) implicit event tracking in the browser, which allowed collecting a large amount of data related to user activity and physical parameters of recommending interfaces. Results have been analyzed in order to compare the reliability and applicability of both methods. Observations made with eye tracking and event tracking led to similar results regarding recommendation interface evaluation. In general, vertical interfaces showed higher effectiveness compared to horizontal ones, with the first and second positions working best, and the worse performance of horizontal interfaces probably being connected with banner blindness. Neural networks provided the best modeling results of the recommendation-driven purchase (RDP) phenomenon.

## 1. Introduction

The continuous growth of e-commerce, especially nowadays due to the coronavirus disease (COVID-19) pandemic, requires tools, particularly recommendation systems that aim to help discover relevant products for individuals. While online shopping benefits generally exceed disadvantages, lack of personal touch, especially when a customer is overwhelmed by many alternative products is perceived as a major obstacle to shopping online. To alleviate this problem, online stores invest in personalization tools, recommendation systems being a very popular example of them. Recommendation adequacy plays a vital role in overall commercial success and customer satisfaction. However, due to the significant loads of marketing content and increasing browsing speeds, users may often not notice the displayed recommendations. Therefore, the way of presenting a recommendation, and its positioning and usability seem to have an important role in the ultimate recommendation effectiveness.

In a personalized recommendation system, a user model is created based on user demographic data and his/her behavior and transactions [1]. A digital representation of this model is a user profile, which usually reflects the user’s preferences and needs. Online websites can collect a broad spectrum of data [2,3,4,5] concerning users’ demographics and behavior, which are used to construct user profiles and generate personalized recommendations aimed to generate sales.

Most popular recommender engines use well-established, collaborative filtering with improvements, e.g., including user interest [6,7]. Another type of content-based recommender engine relies on presenting products similar to products the user liked in the past. The major advantages and disadvantages of each approach are presented in Table 1. Recently, the use of deep neural networks has grown in interest among recommender system researchers [8]. Shahriar et al. (2020) used eye tracking to learn user behavior by counting how many times a user looked at a particular website element in order to generate a cluster of similar products to constitute a base for recommendations [9].

The recommendation system’s performance and effectiveness depend on the underlying recommendation algorithm itself, but it also goes far beyond this point [10]. There is much less research in selecting the best ways the products should be presented to the customer compared to massive research in the field of recommendation algorithms [11,12]. The most appropriate ways of presenting a recommendation item to users can be learned by observing human–computer interaction, with websites and recommending interfaces in particular. While interacting with websites, human behavior can be observed via eye tracking [13] or by collecting events inside modern web browsers [2], based respectively on gaze sensors and mouse/keystroke sensors. The investigation of visual aspects of recommending interfaces could better integrate those interfaces in online stores [10,14]. The position and layout of a recommendation zone, the number of recommendations, the size of associated images, product titles and prices, etc. can all be evaluated toward interface improvement [15]. In the era of information overload, in particular enormous marketing content, the habituation effect often comes into action, resulting in the banner blindness phenomenon [16]. Even the best recommendations from the algorithm perspective may have little effect unless they are properly presented [16,17,18]—with the right layout and position on a website, at the right moment of the browsing and purchase process, with the right visual intensity (understood as the level of content intrusiveness) [19] and in the right context, etc. [20,21,22,23].

This paper is a substantial extension of a conference paper [24], which presented the initial results of an eye-tracking experiment on visual intensity and layout for a simple-structure recommending interface with regard to reducing habituation. The general aim of the study was to evaluate two layouts and three visual intensity levels of item presentation and varied positions of recommendations in a basic-structure recommending interface, while the levels of attention drawn and inspired product interest were monitored.

This paper focuses on the analysis of the effectiveness of recommendation interfaces based on the eye-tracking research in relation to purchasing based on recommendations using a number of machine learning methods. The eye-tracking study results were compared with the results of an implicit feedback study performed via browser add-ons on a group of participants for major e-commerce websites in Poland. The results were analyzed and models for predicting recommendation effectiveness were built using different machine learning methods to find the best-performing ones. This paper’s original contribution also relates to using two different methods of tracking user behaviors and thus user interest—eye tracking and collecting implicit feedback via a web browser add-on—to compare their applicability for building automated mechanisms of recommending interface personalization.

The remainder of the article is structured as follows: Assumptions for the study and methodology are presented in Section 2. The structure and procedures of the experiment are provided in Section 3. Results are discussed in Section 4, and conclusions are presented in Section 5.

## 2. Methodology

The study’s main objective is to verify the effectiveness of recommending interfaces of e-commerce websites with their visual aspects in mind using gaze tracking and human–computer interaction events tracking. Different layouts (vertical versus horizontal) different positions, and varying visual intensities of recommending interfaces were compared with regard to attracting customer interest and recommendation-driven purchase (RDP) from the perspective of user experience and business goals.

While users are usually most interested in the main content of a website, positioning and modifying the intensity of the most accurate recommendations so that they would attract the highest possible attention of users allows for the achievement of various website goals. In turn, that could lead to increased customer satisfaction, retention, long-term increased sale, and finally boosting profits.

User interest can be determined by asking the user explicitly or by observing the interaction implicitly in order to infer it. Unfortunately, explicit questioning may disturb natural behavior as it adds an additional burden on the user [25,26,27]. Furthermore, in the case of fast Internet browsing, there could be a lot of unconscious attraction caused by some webpage parts. Therefore, inobtrusive implicit measures are better suited for the purpose of the study, allowing the monitored subjects to focus only on tasks performed, not causing additional cognitive load, and not requiring unique motivation to provide explicit ratings [28,29].

For the purpose of this study, the results of experiments using various observation techniques have been analyzed. Eye-tracking technology was used for implicit user visual behavior observation and analysis. Eye tracking is the most popular technique of observing human visual activity and providing a large amount of implicit feedback and can be performed with standalone or wearable devices. Within the scope of the study, gaze-based data were used and interpreted in a simple e-commerce scenario. Eye movements were used to discover which areas of a shopping website are mostly looked at, attracting user attention as being the most relevant to the user. Raw data were processed and analyzed with the help of popular eye-tracking software and data analytics algorithms to generate metrics regarding eye fixation and gaze.

Although eye movements are often unconscious and chaotic in nature, they are generally tightly connected with cognitive processes [30]. Thus, based on gaze data from eye tracking, it is possible to conclude about user attention and interest. Gaze is an invaluable source of data for providing information on how much attention a user pays to contents on the screen [31]. For the following analyses, total fixation duration is the main gaze-based measure used. This parameter is used as an indicator of attractiveness by a lot of other studies [32,33,34]. It is calculated as the sum of durations of fixations aggregated on a website section, an area of interest (AOI), one with recommendation content (RC), i.e., presentation of the recommended items, and the main section with editorial content (EC), i.e., the main viewed product description, pictures, etc.

An important property of a recommending interface on a website, in addition to the positioning of recommending interface and recommended items within that interface, is the aspect of visual intensity [35], which is also considered in the study. Changing visual intensity is a popular marketing approach to counteract habituation and attract more attention [35]. Three basic levels of intensity were used.

Another possible technique for implicit monitoring of user behavior on websites is based on human–computer interaction monitoring solutions such as scripts or browser extensions that allow registering events resulting from interacting with the website on the client’s side. With this method, it is possible to observe user behavior unobtrusively without extra attention from them [36] or additional equipment.

Our e-commerce customer preference monitor (ECPM) was built in order to collect e-customer behavior data [2]. This monitoring tool was implemented as an extension for the Mozilla Firefox browser, with the core monitoring code being easily detachable and available for external use. ECPM allows for monitoring of human–website interaction by exploiting document object model (DOM) events. HTML document object model events allow JavaScript to register different event handlers on elements in an HTML document. Our ECPM unobtrusively collects data on visited product pages and user interactions, recording numerous parameters describing the page’s physical attributes and interactions. Among many collected parameters connected with the recommending interfaces were times of mouse cursor spent inside recommendation zones and their physical size measured as the number of characters within recommended products section. As other research shows, mouse position may be quite strongly correlated with eye gaze [37,38,39]. Tracking mouse movements can be used as a lower-quality and easier to perform alternative to eye tracking for the purpose of identification of user interest. Measured times with cursor being over recommended products were related to other measures such as total time spent on the product page and page height and length measured by a number of characters, images, etc. In this way, relative measures of cursor times spent inside particular recommendation interfaces were generated.

## 3. Conceptual System Architecture and Research Experiment Procedure

### 3.1. Conceptual System Architecture

**Figure 1:**
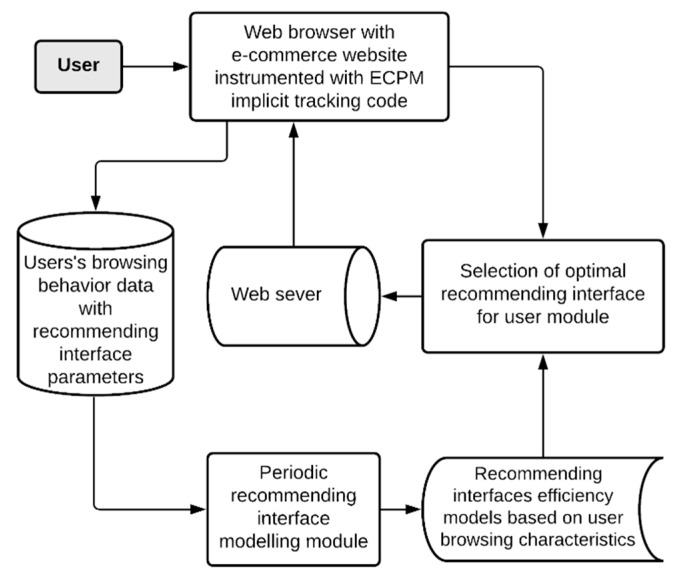
Conceptual system architecture.

The conceptual system architecture is presented in Figure 1. The goal of the system is to adapt recommending interfaces to maximize its efficiency. The core of the system is recommending interface efficiency models based on user browsing characteristics. Adaptation is performed on recognized user behavior characteristics. Those models are generated based on data gathered from all users via implicit tracking of their behavior inside browsers. Current use is matched against models’ database, and the most efficient recommending interface is to be selected.

### 3.2. Research Experiment Procedure

**Figure 2:**
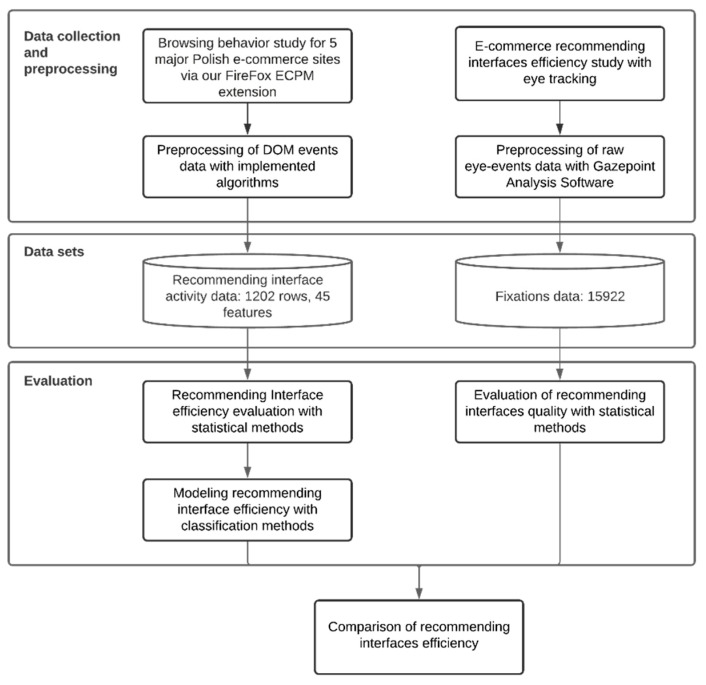
Research experiment architecture.

The research experiment procedure is presented in Figure 2. Results presented in this paper are based on two experiments on recommending interfaces in e-commerce websites, described in detail in the next two sections, one performed with eye-tracking equipment and software on a smaller group of participants, and the other performed using our own implicit browsing behavior monitoring tool in the form of a web browser extension—on a larger group of participants. Both experiments used simple e-commerce scenarios where participants were browsing products and expressing interest in them. The main goal was to analyze recommending interface efficiency for two groups of users with different ways of collecting data about users’ activity. Results from both methods have been cross-compared in order to estimate their applicability for automatic recommending interface personalization in e-commerce scenarios, but not limited to them.

**Figure 3:**
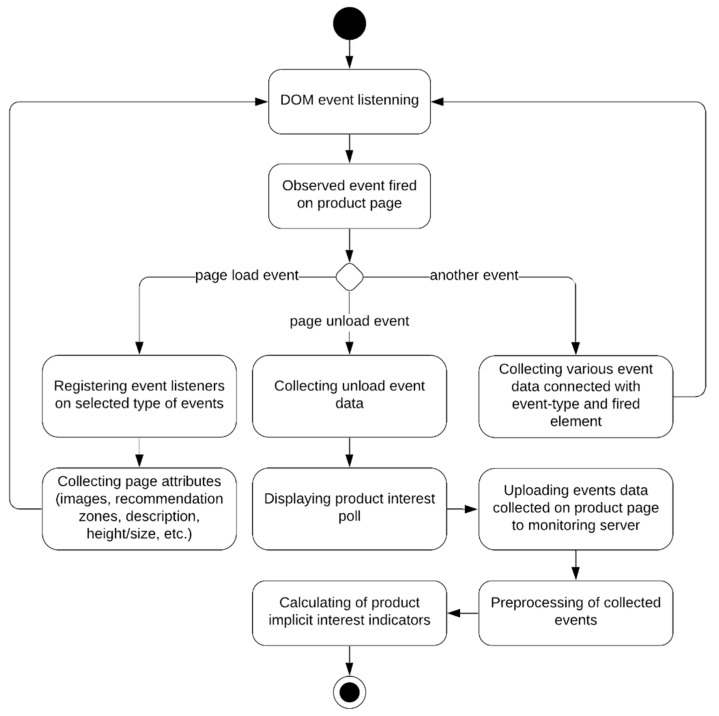
Activity diagram depicting implicit data processing with the behavior monitoring tool.

Figure 3 shows the unified modeling language (UML) activity diagram depicting implicit data processing with our ECPM behavior monitoring system. Three decision paths exist between the start point and the finish point while the activity is being executed for different types of events.

### 3.3. Eye-Tracking Experiment Structure and Procedure

This section describes the experiment performed to collect behavior data, which were used to train five different classifiers responsible for evaluating of recommending interfaces. The experimental group of users consisted of 52 people, 14 women and 38 men, all of whom produced valid eye-tracking data. Most of them were attracted by advertisements for the study or invited in person. They ranged in age from 14 years old to 54 years old (mean = 25.2, σ = 8.0). One limitation of the study was the large difference in the ratio of women to men and the big age span, although 90.4% of the participants were 18–39 years old. The group of subjects taking part in the study was not randomly selected and did not constitute a representative subset of the whole population.

The study design was within subjects. All participants were given the same task—to shop online to furnish a studio apartment with six kinds of furniture fully. A subject was asked to go from one product category to another and choose an item from each category. For the purpose of the experiment, a dedicated e-commerce website was developed using Drupal CMS. The website was composed of a recommending interface. Each participant was provided with the same website and the same product sets. The pages were written in Polish and consisted of the title, menu, product images, and descriptive text. The website covered functions such as product list, buying cart, and recommendations.

The editorial content was placed in the central part of the screen, under the main menu, and included product lists that were approximately three-screen pages long and contained 10 products for each product category. Each product had three unique features—name, an image of the product, and price. Products in a category were quite similar visually and similarly priced. There were six product categories PC*j*— wardrobes, chests of drawers, beds, bedside cabinets, tables, and chairs. Moreover, an “Add to Cart” button was placed under the description of a piece of furniture allowing to store customer selections in a database. From the moment a product is selected, its short description is available in cart preview and the main cart page. It is possible to remove a product from the cart in order to allow a user to make necessary changes to the final selection.

**Figure 4:**
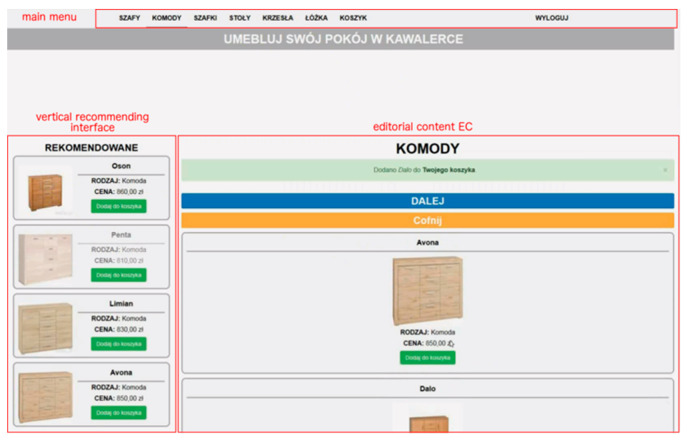
Vertical layout of the recommending interface.

**Figure 5:**
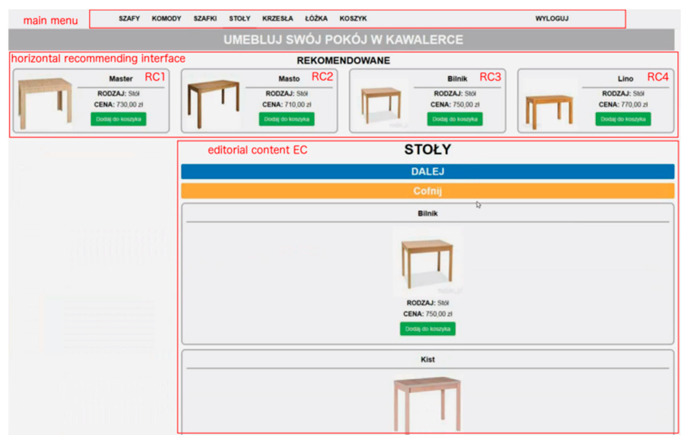
Horizontal layout of the recommending interface.

There were two alternative recommendation modes, i.e., horizontal and vertical recommending interface layout, which means that the RC section was anchored in one of two dedicated parts of the screen below the main menu, either on the left side of the page next to the general product list (vertical mode) or at the top of the page, above the general product list (horizontal mode). Only one recommendation layout was available at one time, i.e., when the horizontal mode was on, the vertical one was deactivated and vice versa. Figure 4 and Figure 5 show two alternatives to the general location of the recommended content.

The RC section always consisted of four recommendation items, RC1 to RC4, randomly selected from all products in a given category. The section in each variant did not change its location on the screen throughout browsing products in a product category, regardless of the EC section’s user scroll. Only the general product lists were actually scrollable in order to ensure reliable subject exposure to recommendations.

**Figure 6:**
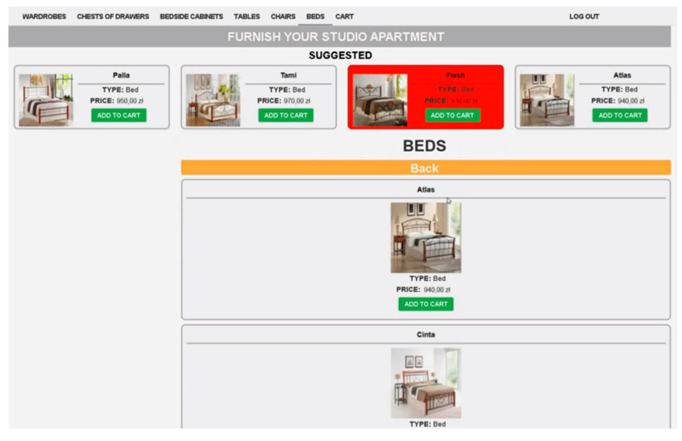
Example of visual intensity VI3 (red background) in the vertical recommending interface.

It was ensured that product features, i.e., name, image, and price did not stand out from other products in a category. It was intended that the distinction of a particular RC*i* location would be accomplished only by means of visual intensity (VI). Three levels of intensity were used—standard (no highlight) VI1, flickering (slowly disappearing and reappearing every 1–2 s) VI2, and background in red VI3. For each product category, there was a maximum of one RC*i* at VI2 or VI3. An example of the visual intensity of the last kind (VI3) is presented in Figure 6.

An experimental run proceeded in a way that the examined person was explained what the device for sensing eye movements was, and the eye tracker was calibrated using Gazepoint Control software (Gazepoint, Vancouver, BC, Canada) and a nine-point calibration method. For better accuracy, the calibration was always performed twice the first time to familiarize the subject with the process. There was a double monitor setup, and the operator’s screen was invisible to the participant. Due to the correct calibration, it was possible to determine precise coordinates of the place the user was looking at. We did not employ data loss measures because in these short tasks with a given goal, the data loss rate is very low. To overcome any data loss, calibration was performed twice, as mentioned earlier. All recordings from the eye tracker were also carefully analyzed in order to confirm the proper performance of the experiment. There was no eyewear that could interfere with pupil tracking.

Each participant was informed what their task was, but they were not told about the study’s goal yet. After this introduction, the subject had to furnish the apartment. After choosing one item from a category, the subject clicked “Next” and was automatically moved to the next category. Category after category, the visual intensity of recommendation items changed each time, following the same order for each participant. Furthermore, for the first three categories, the RC layout was vertical. After moving to the fourth category, it changed to horizontal and remained this way for the following categories. On the whole, there were at least six subsequent pages with different recommendation variants presented to each participant.

The eye-tracker device used was Gazepoint GP3 (Gazepoint, Vancouver, BC, Canada) with a 60 Hz update rate (satisfactory taking into account the nature of the research and observing fixation times), the device’s nominal accuracy being 0.5–1° of visual angle, the range of depth movement ±15 cm, calibration 5- and 9- point. Every session was recorded and controlled live using Gazepoint Analysis 4.3.0 software (www.gazept.com (accessed on 16 February 2021)), and it was double-checked continuously on the operator’s monitor that the experimental setup was correct. After completing the task by the participant, basic data such as age were collected, and a question was asked whether a subject felt recommendations influenced them. Finally, all data were saved and stored for further analysis. A typical experimental run lasted around 12 min.

Results from Gazepoint Analysis software have been preprocessed with a simple algorithm whose goal was to calculate total fixation time while the user gazed at particular areas of interest. The fixation point-of-gaze (POG) coordinates data FPOGX and FPOGY, which provide the user’s point-of-gaze as determined by the internal fixation filter, have been used together with information about the location of particular sections (e.g., recommending interface elements) of analyzed websites. Based on those data, the total time while the user was actively gazing at particular elements of the website was calculated. Only valid fixation POG data points (flag FPOGV) have been counted. It needs to be noted that some gaze tracking signals may occur in the absence of any specific state. The nature of gaze itself is variable due to breathing, the gravity of expressed interactions, etc.

### 3.4. Implicit Event Tracking via ECPM Browser Extension

The ECPM tool was instrumented for five major Polish online stores: Merlin.pl, Agito.pl, Electro.pl, Komputronik.pl, and Morele.net. From the perspective of assortment at the time of the study, Agito.pl and Merlin.pl were horizontal shops, whereas Electro.pl and Morele.net offered mostly electronic goods. Merlin.pl was a major Polish online bookstore.

All 85 participants for the study were non-profit volunteers who were active web users located in Poland, aged between 19 years old and 33 years old, all holding at least a high school degree. The task given to participants was to browse for interesting products and rate them. After leaving the product page, a rating form was displayed, where the user could express his/her level of being pleased by the product. User activity was monitored at the most granular level. Every DOM-fired event connected with the mouse and keyboard was registered together with detailed information about the source element and its position in the structure of a web page. In addition, the HTML code of every visited product page was collected. Those data were used to generate parameters describing user behavior, which were classified into four groups—describing product page attributes, describing user interaction times (in ms), describing user behavior per se, and also relative parameters describing user behavior.

During the study, 1396 products were rated, and all customer interactions with websites were monitored via ECPM. About half of the participants rated below 14 products, while the upper quartile rated more than 20 products. Higher ratings were more popular than lower ones. Among many parameters, ECPM monitored user interaction with recommending interfaces. The main monitored measure was the total time while the mouse pointer was positioned over other recommended products section. This measure was used to reflect user interest in the recommendation section.

### 3.5. Comparing Visual Aspects of Recommending Interfaces

This section describes the procedure employed to compare visual aspects of the recommending interfaces for observations made with eye tracking and those based on DOM events. The size and resolution of the screen were mostly the same for both studies. The eye-tracking study was conducted in a university laboratory on the same equipment. The implicit tracking with ECPM was conducted in a university laboratory and at personal computers of volunteers taking part in the study from home. The resolution and size of the screen were recorded in the study and were the same or very similar for different participants.

Data collected using the eye-tracking device were used to build a classification model with five different classification modeling methods. Gazepoint Analysis software was used to gather fixations made by participants and process them into lines of data. Overall, 15,922 fixations lines of records have been stored. Those records have been pre-processed to generate features appropriate for analysis and classification modeling. Every row of the resulting dataset contained features shown in Table 2. Only behavior-related parameters were used, without any demographic data, compared to previous studies [24,40].

As a measure of recommendation efficiency add-to-cart actions were used, being the most appropriate due to the selection and purchase task given to participants. The pre-processed data from the eye-tracking study were used for building classification models using five different modeling methods—logistic regression, decision trees, random forests, neural networks, and support vector machines. Those methods were selected as being commonly used and well established [41,42]. Our goal was to compare those methods in terms of classification quality in order to select the most suited one for the task of evaluating recommending interfaces.

The whole data set was split into training and testing sets with a proportion of 70% to 30% with stratified sampling, which resulted in roughly the same proportion of each class cases between both training and test sets. The testing set was used to evaluate models’ metrics. The training was performed with selected values for the hyper-parameters presented in Table 3.

Detailed data collected with ECPM were used to extract parameters concerning user behavior in recommendation zones together with features of the recommending interface and product pages. Every row contained the following features presented in Table 4.

Those parameters were used to compare the effectiveness between different recommendation layouts—horizontal and vertical. As a measure of effectiveness, the time of the mouse being positioned over the recommending interface was used as the most appropriate due to the goal that was given to participants of the study. This measure was related to different physical attributes of the page. Komputronik.pl store was omitted from the analysis, in opposition to previous studies, because it contained only vertical recommending interfaces.

Finally, results from the experiments were compared in order to find similarities or dissimilarities in evaluations of recommending interfaces.

## 4. Results and Evaluation

### 4.1. Eye-Tracking Results of Recommending Interface Efficiency Evaluation

Analysis of data collected with eye trackers shows that finishing the task averagely took 2.3 min. Overall, 312 products were chosen for purchase in the preliminary study. The time of fixation on the recommending interface was on average 16.3 s per participant, which constitutes 12% of the mean task finishing time. Subjects devoted an averagely of 8.2 s for observing vertical RC and 8.1 s for horizontal RC. This means that two presented layout variants of the recommending interface (horizontal and vertical) performed equally in fixation times.

The distribution of fixation times for all recommendation item positions is shown more exhaustively in Table 5. The first three RC*i* positions were most favorable regardless of the layout type, whereas the fourth positions in the list were the least eye-catching ones. Surprisingly, the most popular of all was the RC3 location in a horizontal layout (3.9 s). This might have been affected by the fact that this recommendation item was located right above the main product list. Next in terms of popularity were sections RC2 and RC1, respectively, situated in the vertical layout. The clear popularity of RC2 in this layout was caused by the flickering (VI2) effect shown in one product category. The popularity of RC1 although always shown without any extra visual intensity effects (VI1) may be resulting from that many people tend to perceive the first/top location in a list being the best one. In the case of the vertical layout, the first location performed better than RC3 despite presenting it with very strong intensity VI3 for one product category. Both items RC3 in the vertical layout and RC2 in the horizontal layout performed equally despite that the latter was distinguished with flickering effect (VI2) for one product category.

From the sales view, 12% of products were added to carts directly from the recommending interfaces, while around 13% of products were firstly seen by subjects in RC and added from the main EC. This constitutes roughly 25% of all products being added to the cart directly from RC or indirectly (influenced by RC). Around 75% of products were selected directly from the main EC without any influence from RC. Coincidentally, it is exactly the same proportion as the one of recommending interface fixation time to task completion time, which somehow shows the importance of focusing attention on recommended items. Vertical RC layout accounted for 60% of adding to cart actions, while the rest were thanks to horizontal RC layout—the vertical one turned out to be much more effective than the horizontal. This may connect with the so-called banner blindness phenomenon, where banners have often been located in the same site area as horizontal recommendations in the analyzed experiment.

The vertical layout resulted in a much better rate of adding products to the cart despite a similar average time of gaze for both interfaces. It is presumed that this may be caused by the fact that the horizontal recommending interface was located at the top of the page directly under the main menu. This location of the menu required often forced unintentional gaze at recommending interface. Initial analysis of eye-tracking video recordings confirmed this hypothesis in many cases. After adding products from vertical RC or main EC, users were often stopped at horizontal RC before selecting the next category from the menu.

In the case of the vertical layout, for RC with all RC*i* at standard intensity level (VI1), the purchases directly or indirectly driven by recommendations (RDPs) were equally distributed between recommended products; for RC with RC2 at a flickering intensity level (VI2), the item attracted 7 out of 18 RDPs in the product category; for RC with RC3 in the red background (VI3), the item attracted surprisingly only 2 out of 15 RDPs in the product category. Overall, RC2 performed best, which means that the second recommendation in the vertical list generally resulted in the biggest sales (43% of RDPs for vertical RC, and 35% of all RDPs). The volumes of recommendation driven purchase per participant are presented more exhaustively in Table 6.

Additional side remark resulting from the visual analysis of fixations paths shows that the flickering effect (VI2) of a recommendation item appeared to have an extended effect on fixation after moving to the next product category, which means that despite the visual intensity reverted to standard this recommendation location continued to attract more attention of users.

After completing the task, a survey was conducted, and 33% of the participants responded that they felt their selections were affected by recommendation content (6% felt strongly about it). In contrast, the rest responded that they felt the opposite, including 52% who claimed definitely that they ignored recommendations. This last group showed strong resistance to recommendations—when presented the RC elements after the test, some were surprised to see they had probably neglected most of them, taking them for unwanted advertisements, which suggests the effect of habituation occurred.

### 4.2. Modeling Recommending Interface Efficiency Based on the Eye-Tracking Experiment

Five different classification methods were used to model the recommending interface efficiency based on add to basket actions. Models were built separately for three different target variables, namely, *add_to_cart_direct*, *add_to_cart_indirect*, and *add_to_cart_both*. Model validation techniques and hyper-parameters used for training various classifiers have been presented in Table 3.

#### 4.2.1. Classification Accuracy for Target Parameter *add_to_cart_both*

For target parameter *add_to_cart_both*, ranking of variables with Gain ratio and Gini is mostly similar showing that most valuable variables are *fixation_time_location*, *share_time_location_category*, *rc_location_intensity*, *fixation_time_location_layout*, and *share_time_layout_category*. On the opposite side, variables with the lowest ranks are *fixation_time_category*, *rc_location*, and *rc_layout*.

All models targeting the *add_to_cart_both* parameter have very good precision measures with accuracy classification score (CA) above 90% for all models and area under the curve (AUC) having high values for all models except Binary Tree (Table 7).

All models obtained for the target parameter being *add_to_cart_both* with accuracy measured as average over classes have relatively high precision with Neural Network performing best (Table 8).

For the target class being 1, which denotes the action of direct or indirect addition to cart, all models have lower precision compared to models with accuracy measured as average over classes. However, precision and specificity are still very high (Table 9).

For the target class being 0, which denotes that the product was not added to the cart directly or indirectly from the recommending interface but was added from editorial content without registering gaze at this product within the recommendation zone. These models have the highest classification precision of all three types of models meaning that models are performing best in predicting not adding product to basket recommending interface directly or indirectly (Table 10).

#### 4.2.2. Classification Accuracy for Target Parameter *add_to_cart_direct*

For the target parameter *add_to_cart_direct*, a ranking of variables with Gain ratio and Gini is mostly similar to where *add_to_cart_both* was denoted as the target variable. The only difference is the lower rank of *rc_location_intensity*. General precision metrics, both AUC and CA, for all models are very high showing their outstanding capabilities (Table 11).

With accuracy measured as average over classes, all models have precision above 93% with the Binary Tree, also having the highest specificity (Table 12).

For the target class being 1, which denotes the action of direct addition to cart from the recommending interface, all models have much lower precision compared to models with accuracy measured as average over classes (Table 13). Again, Binary Tree is the best with 69.2% precision.

Precision for not adding product to cart directly or indirectly from recommending interface is again higher for all models (Table 14).

#### 4.2.3. Classification Accuracy for Target Parameter *add_to_cart_indirect*

For the target parameter *add_to_cart_indirect,* a ranking of variables with Gain ratio and Gini is mostly similar to other target variables. The only difference is the higher rank of *rc_location_intensity*.

Models for indirectly adding products to cart have different accuracy classification scores with Random Forests and Neural Network having the highest values (Table 15).

With accuracy measured as average over classes, all models have precision above 86%, with Neural Network having the highest value (Table 16).

For the target class being 0, which means that products have been seen only on the main EC and added to cart from that place, all models have relatively high precision (Table 17).

For the target class being 1, which means that the user gazed at a product before adding it to the cart from EC, all models have relatively low precision from 52% to 64.8% (Table 18).

**Figure 7:**
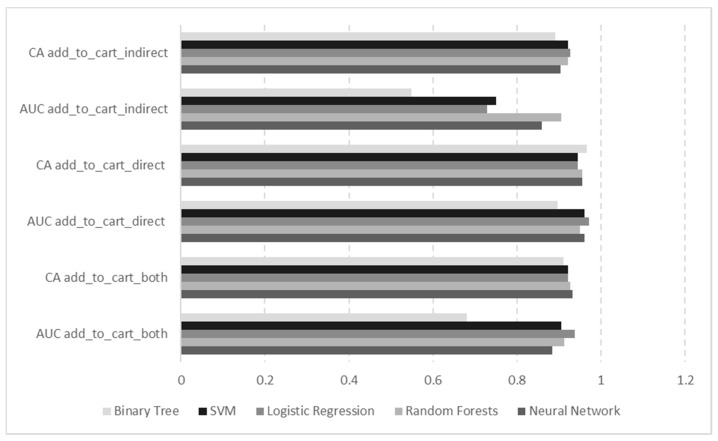
Classification accuracy metrics for all target variables received for different models.

Classification accuracy metrics (AUC, CA) for all three target variables obtained for different models are presented for easier comparison in Figure 7.

### 4.3. Results of Recommending Interfaces Efficiency Evaluation Based on the Event Tracking Study

Comparing recommending interfaces efficiency based on data gathered during our ECPM-based experiment shows that average *prod_recommend_time* was significantly higher for vertical layout than horizontal layout in case of three of four shops (Table 19).

Only Morele.net was in the opposite, which may well be caused by a higher number of horizontal recommending zones than vertical (two versus one) in this shop. The parameter that shows the level of interest in recommendation *rel_recommend_time_recommend_length* compared to its length proves that vertical zones gained more attention than horizontal ones. Other relative parameters presented in Table 19 confirm this tendency.

## 5. Conclusions

Recommender systems are commonly used to draw customers’ attention to products being consistent with their preferences. They are expected to result in higher conversion rates, purchase volumes, and client retention. This study showed the influence of the recommending interface on user behavior in simply structured and real-world e-commerce stores. By combining evaluation based on two different methodologies—eye tracking and implicit behavior event tracking inside the browser, which is an original contribution of this paper—a large amount of data related to user activity and physical parameters of recommending interfaces were collected. Results were analyzed in order to compare the reliability and applicability of both methods, and the classification quality of popular modeling methods was verified with regard to recommending interfaces evaluation.

In the eye-tracking experiment, an average of 12% of task completion time was devoted to looking at the recommending interfaces, and the same percentage of goods were bought with direct use of recommendations. Comparing the vertical and horizontal recommending interface layouts, they performed equally in terms of fixation times. Still, from the perspective of purchase commitments, the vertical layout turned out to be almost twice as effective as the horizontal. In the better performing vertical layout, the most attractive were the first and second positions in the list. A slow flickering effect used in the second position influenced its attractiveness. On the other hand, the high visual attractiveness of the first position in the list, despite the lack of any visual highlight, may have resulted from the conception that the first one is always the best (similarly to search results). The worse effect of the horizontal layout may be connected with the so-called banner blindness. The event tracking experiment also showed the higher attractiveness of vertical zones compared to horizontal ones. To the best of our knowledge, this is the first study that verified this result using different methods and data sources.

Various classification models for predicting actions of adding product to cart directly from recommending interface or indirectly (influenced by displayed recommendation – gaze tracked) or any of those actions show overall very good precision. The accuracy measured as average over classes for all models with target variable *add_to_cart_both* varied in precision from 90.1 to 92.9%, with Neural Network performing best. The accuracy of predicting adding product to cart exclusively directly or indirectly from recommending interface ranged from 55.6 to 92.6% for precision metric, again with Neural Network performing best. Generally, good quality was provided with models based on Neural Network, Logistic Regression, and Random Forests.

The study showed that both approaches, gaze and event-tracking based, showed similar results in terms of recommendation interface presentation evaluation, and as such, they are similarly applicable for building automated mechanisms of recommending interface personalization and improving human–computer interaction. Both techniques confirmed the higher efficiency of vertical recommending interfaces compared to horizontal ones. Users spent relatively more time looking at vertical recommending interfaces, which resulted in more interactions related to adding products to the cart. The recommendation position inside the recommending interface proved to play an important role in a good performance for middle positions—second for vertical and third for horizontal layout. The visual intensity had a varied influence with the flickering effect attracting most users.

The study had some limitations, in particular, the subjects in the study were not randomly selected and were not a representative subset of the whole population. They, however, provided cognitive value in the context of the effectiveness of tracking techniques and machine learning methods for identifying user behavior indicative of purchase intent.

In future research, we intend to extend the study to a larger and statistically significant scale by partnering with a growing number of e-commerce stores and leveraging the usage of implicit activity tracking to receive the ultimate benefit of recommendation optimization. We will use the most promising modeling methods. In our experiments, we have not dealt with eye fatigue and eye–hand coordination, which can affect the results’ reliability [43,44,45,46], nor with the relation of gaze tracking to the cognitive realm, which is a significant part when deciding whether to purchase or not. Therefore, we plan to use more advanced eye tracking for gathering more data, considering the variable nature of gaze and detecting eye fatigue issues. We also intend to include the cognitive emotion information based on the fact that different types of eye motions can represent different emotions [47,48] and integrate a facial expression recognition (FER) module to include data on human sentiment while surfing the e-commerce sites. We will also perform both eye tracking and implicit events tracking at the same time to cross-compare their results and possibly come up with an optimized hybrid approach. Moreover, we want to develop our events-based solutions to recognize users’ activity, including time spent displaying particular AOIs in recommending interfaces on computers and mobile devices, focusing on visual aspects such as layout and intensity, in order to seek best-performing solutions for this multidimensional problem.

## Figures and Tables

**Table 1:** Main recommendation techniques—advantages and disadvantages.

Recommendation Technique	Advantages	Disadvantages
Collaborative filtering	Serendipity—user can discover new items/products Intuitive and rationale recommendations Easy scalability No need for content data about items/products	Cold start problem for both new users and new items Low recommendation quality for sparse data Model training can be expensive
Content-based	User independence—the model needs data only about the current user Explainability of the results Ability to generate a recommendation for the user with a unique taste	Lack of recommendation novelty Over-specialization Cold start problem for both user and item Requires description of products/items

**Table 2:** Features generated based on eye-tracking device.

Feature	Feature Description
*rc_layout*	RC layout (horizontal/vertical)
*rc_location_intensity*	intensity level of a recommendation location (1–3)
*rc_location*	RC*i* position (1–4)
*fixation_time_location*	overall fixation time for RC*i* position
*fixation_time_layout*	overall fixation time for RC layout
*fixation_time_category*	overall time spent by the participant on a product category page
*share_time_location_category*	fraction of time when fixation was registered inside RC*i* position with respect to the overall time spent on a category page
*share_time_layout_category*	fraction of time when fixation was registered inside RC layout to the overall time registered on a product category page
*share_time_location_layout*	proportion of time when fixation was observed inside RC*i* position to the overall time registered on RC layout
*add_to_cart_direct*	action of adding the product to cart directly from RC
*add_to_cart_indirect*	action of adding the product to cart action from EC while product was looked at in RC
*add_to_cart_both*	adding the product to cart action from RC or EC while product was being observed in RC

**Table 3:** Values of classification model hyper-parameters.

Model	Hyper-Parameter	Hyper-Parameter Value
Neural Network	activation function	Rectified Linear (ReLU)
Neural Network	maxium number of neurons in hidden layer	150
Decision trees	stop extending a tree when majority reaches	95%
Decision trees	do not split subsets smaller than	5
Logistic Regression	regularization function	Ridge (L2)
Logistic Regression	strength	C = 0.18
Random Forests	maxium number of trees	15
Random Forests	depth limit of individual tree	3
Random Forests	splitting stop subset limit	5
SVM	kernel	RBF with Auto Gamma
SVM	cost	1
SVM	regression loss epsilon	0.1

**Table 4:** Features generated based on data gathered via the e-commerce customer preference monitor (ECPM) monitoring tool.

Feature	Feature Description
*rc_layout*	RC layout (horizontal/vertical)
*recommend_length*	number of characters within all recommended products sections
*document_length*	number of characters within all texts on the page
*page_time*	time between page load and page unload
*tab_activ_time*	time while tab containing particular page is active
*user_activ_time*	time while user is actively interacting with page (generating keyboard or mouse events)
*rc_prod_recommend_time*	time while mouse pointer was positioned over recommended products
*rel_recommend_time_document_length*	relative time while mouse pointer was positioned over recommending interface in relation to the number of characters within all texts on the page
*rel_recommend_time_page_time*	relative time, while mouse pointer was positioned over recommending interface, in relation to time between page load and page unload
*rel_recommend_time_tab_activ*	relative time while mouse pointer was positioned over recommending interface in relation to time while tab containing particular page is active
*rel_recommend_time_user_activ*	relative time while mouse pointer was positioned over recommending interface in relation to time while user is actively interacting with page (generating keyboard or mouse events)

**Table 5:** Average fixation time (s) for each recommendation location.

Recommendation Location	Time (s)	
	Vertical RC	Horizontal RC
RC1	2.4	1.3
RC2	3.1	2.1
RC3	2.1	3.9
RC4	0.6	0.8
Total	8.2	8.1

**Table 6:** Recommendation-driven purchase and visual intensity for each recommendation location RC*i* and product category PC*j*.

Recommendation Location	Vertical RC			Horizontal RC		
	PC1	PC2	PC3	PC4	PC5	PC6
RC1 direct	0.019 (VI1)	0.019 (VI1)	0 (VI1)	0.038 (VI1)	0 (VI1)	0.077 (VI1)
RC1 indirect	0.019 (VI1)	0 (VI1)	0 (VI1)	0.019 (VI1)	0 (VI1)	0.058 (VI1)
RC2 direct	0.038 (VI1)	0.077 (VI2)	0.096 (VI1)	0 (VI1)	0.058 (VI2)	0 (VI1)
RC2 indirect	0.038 (VI1)	0.058 (VI2)	0.077 (VI1)	0.019 (VI1)	0.038 (VI2)	0.019 (VI1)
RC3 direct	0.019 (VI1)	0.077 (VI1)	0.019 (VI3)	0 (VI1)	0.019 (VI1)	0.038 (VI3)
RC3 indirect	0.058 (VI1)	0.096 (VI1)	0.019 (VI3)	0 (VI1)	0.038 (VI1)	0.058 (VI3)
RC4 direct	0.038 (VI1)	0 (VI1)	0.038 (VI1)	0 (VI1)	0.038 (VI1)	0 (VI1)
RC4 indirect	0.019 (VI1)	0.019 (VI1)	0.038 (VI1)	0 (VI1)	0.058 (VI1)	0.019 (VI1)
**Total direct**	**0.115**	**0.173**	**0.154**	**0.038**	**0.115**	**0.115**
**Total indirect**	**0.135**	**0.173**	**0.135**	**0.038**	**0.135**	**0.154**

**Table 7:** Models accuracy metrics for *add_to_cart_both* as target parameter.

Metric	Neural Network	Random Forests	Logistic Regression	SVM	Binary Tree
AUC	0.883	0.913	0.938	0.905	0.681
CA	0.932	0.927	0.921	0.921	0.910

**Table 8:** Models accuracy metrics average over classes with *add_to_cart_both* as target parameter.

Model	F1	Precision	Recall	Specificity
Neural Network	0.929	0.929	0.932	0.694
Random Forests	0.919	0.923	0.927	0.582
Logistic Regression	0.911	0.916	0.921	0.544
SVM	0.911	0.916	0.921	0.544
Binary Tree	0.901	0.901	0.910	0.543

**Table 9:** Models accuracy metrics for add to cart action (class 1) with *add_to_cart_both* as target parameter.

Model	F1	Precision	Recall	Specificity
Random Forests	0.649	0.857	0.522	0.987
Logistic Regression	0.611	0.846	0.478	0.987
SVM	0.611	0.846	0.478	0.987
Neural Network	0.714	0.789	0.652	0.974
Binary Tree	0.579	0.733	0.478	0.974

**Table 10:** Models accuracy metrics for not adding a product from recommending interface (class 0) with *add_to_cart_both* as target parameter.

Model	F1	Precision	Recall	Specificity
Neural Network	0.962	0.949	0.974	0.652
Random Forests	0.959	0.933	0.987	0.522
Logistic Regression	0.956	0.927	0.987	0.478
SVM	0.956	0.927	0.987	0.478
Binary Tree	0.949	0.926	0.974	0.478

**Table 11:** Models accuracy metrics for *add_to_cart_direct* as target parameter.

Metric	Binary Tree	SVM	Random Forests	Neural Network	Logistic Regression
AUC	0.897	0.961	0.950	0.960	0.971
CA	0.966	0.944	0.955	0.955	0.944

**Table 12:** Models accuracy metrics average over classes with *add_to_cart_direct* as target parameter.

Model	F1	Precision	Recall	Specificity
Binary Tree	0.967	0.969	0.966	0.828
SVM	0.941	0.939	0.944	0.487
Random Forests	0.955	0.955	0.955	0.657
Neural Network	0.953	0.951	0.955	0.573
Logistic Regression	0.941	0.939	0.944	0.487

**Table 13:** Models accuracy metrics for add to cart action (class 1) with *add_to_cart_direct* as target parameter.

Model	F1	Precision	Recall	Specificity
Binary Tree	0.750	0.692	0.818	0.976
SVM	0.500	0.556	0.455	0.976
Random Forests	0.636	0.636	0.636	0.976
Neural Network	0.600	0.667	0.545	0.982
Logistic Regression	0.500	0.556	0.455	0.976

**Table 14:** Models accuracy metrics for not adding product from recommending interface (class 0) with *add_to_cart_*direct as target parameter.

Model	F1	Precision	Recall	Specificity
Binary Tree	0.982	0.988	0.976	0.818
SVM	0.970	0.964	0.976	0.455
Random Forests	0.976	0.976	0.976	0.636
Neural Network	0.976	0.970	0.982	0.545
Logistic Regression	0.970	0.964	0.976	0.455

**Table 15:** Models accuracy metrics for *add_to_cart_indirect* as target parameter.

Model	Neural Network	Binary Tree	Logistic Regression	Random Forests	SVM
AUC	0.858	0.548	0.729	0.905	0.750
CA	0.904	0.891	0.927	0.921	0.921

**Table 16:** Models accuracy metrics average over classes with *add_to_cart_indirect* as target parameter.

Model	F1	Precision	Recall	Specificity
Neural Network	0.902	0.900	0.904	0.562
Binary Tree	0.884	0.886	0.881	0.526
Logistic Regression	0.897	0.869	0.927	0.508
Random Forests	0.894	0.868	0.921	0.496
SVM	0.894	0.868	0.921	0.446

**Table 17:** Models accuracy metrics for add to cart action (class 1) with *add_to_cart_indirect* as target parameter.

Model	F1	Precision	Recall	Specificity
Neural Network	0.949	0.926	0.952	0.616
Binary Tree	0.936	0.919	0.933	0.562
Logistic Regression	0.962	0.912	0.994	0.562
Random Forests	0.959	0.891	0.988	0.568
SVM	0.959	0.891	0.988	0.538

**Table 18:** Models accuracy metrics for not adding product from recommending interface (class 0) with *add_to_cart_indirect* as target parameter.

Model	F1	Precision	Recall	Specificity
Neural Network	0.795	0.648	0.768	0.424
Binary Tree	0.786	0.646	0.751	0.418
Logistic Regression	0.698	0.562	0.717	0.403
Random Forests	0.657	0.520	0.626	0.387
SVM	0.657	0.520	0.626	0.340

**Table 19:** Recommending interface quality parameters based on the ECPM study.

Store/*rc_layout*	*prod_recommend_time* [ms]	*rel_recommend_time_* *recommend_length* [ms/char]	*rel_recommend_time_* *document_length* [ms/char]	*rel_recommend_time_* *page_time*	*rel_recommend_time_* *tab_activ*	*rel_recommend_time_* *user_activ*
Agito.pl vertical	2669	1.584	0.075	0.059	0.067	0.101
Agito.pl horizontal	1920	1.304	0.054	0.043	0.048	0.073
Electro.pl vertical	4029	2.048	0.157	0.089	0.096	0.174
Electro.pl horizontal	1858	0.944	0.072	0.041	0.044	0.080
Merlin.pl vertical	1977	0.351	0.108	0.049	0.084	0.156
Merlin.pl horizontal	1312	0.285	0.072	0.032	0.056	0.104
Morele.net vertical	1777	0.905	0.104	0.054	0.052	0.079
Morele.net horizontal	1953	0.752	0.114	0.059	0.058	0.087

## Data Availability

Some data presented in the study are available from the corresponding author, upon request, data sharing being limited due to personal information content, and the code used during the study being proprietary.
